# Double-Positive Anti-GBM and ANCA-MPO Vasculitis Presenting With Crescentic Glomerulonephritis

**DOI:** 10.7759/cureus.14806

**Published:** 2021-05-02

**Authors:** Mariana Pacheco, João E Silva, Clara Silva, Neuza Soares, Jorge Almeida

**Affiliations:** 1 Internal Medicine, Centro Hospitalar Universitário de São João, Porto, PRT

**Keywords:** anti-gbm disease, mpo-anca vasculitis, crescentic glomerulonephritis, overlap anca and anti-gbm, acute renal injury, imunossupression

## Abstract

We report the case of a 61-year-old man with rapidly progressing glomerulonephritis (RPGN) due to double-positive anti-neutrophil cytoplasmic antibodies (ANCA) and anti-glomerular basement membrane antibodies (GBM) vasculitis. The past medical history included stable untreated psoriatic arthritis and arterial hypertension. He presented with asthenia, anorexia, and rapidly deteriorating renal function with metabolic acidosis and hyperkalemia evolving with the need for hemodialysis. No nephrotoxic drugs were identified. Urinalysis showed proteinuria, erythrocyturia, and mild leukocyturia with no pathological casts and renal ultrasound excluded obstruction as the cause of the acute kidney injury. The subsequent study established the diagnosis of double-positive ANCA and anti-GBM vasculitis with renal biopsy confirming the presence of crescentic glomerulonephritis. The patient was started on corticosteroids, cyclophosphamide, and plasmapheresis with the improvement of symptoms and decrease of antibody titers. The renal function recovery was not obtained and referral for transplantation is ongoing.

## Introduction

Both anti-glomerular basement membrane (GBM) disease and anti-neutrophil cytoplasmic antibodies-myeloperoxidase (ANCA-MPO)-associated vasculitis are small vessel vasculitides that can precipitate the development of rapidly progressive glomerulonephritis (RPGN) and diffuse alveolar hemorrhage (DAH). These are both rare entities that confer an increased risk of organ functional loss and mortality from respiratory or renal failure [1,2].

Despite the rareness of each of these individual entities, the occurrence of overlap ANCA and anti-GBM displays a higher frequency than would be dictated by chance and it might determine disease onset and course. Up to half of the individuals with anti-GBM disease present, double-positivity and approximately 10% of patients with ANCA vasculitis have positive titers of anti-GBM antibodies [1-3]. 

Anti-GBM disease is very rare, with an incidence under two cases per million people per year, and it’s associated with anti-GBM antibody deposition in the basement membrane of the glomerulus [1]. 

Renal involvement in anti-GBM disease presents as acute or subacute kidney injury. Urinalysis can show proteinuria and active sediment with dysmorphic red cells, leukocytes, and pathological casts. In anti-GBM disease, macroscopic hematuria is more commonly present when compared with other rapidly progressive glomerulonephritis. Renal involvement can vary widely in severity [4]. 

Pulmonary involvement in anti-GBM disease presents generally as alveolar hemorrhage and, in rare cases, it can be the predominant feature. Patients complain of dyspnoea, cough, and/or hemoptysis. Pulmonary infiltrates can appear on the chest radiograph and the carbon monoxide diffusing capacity (DLCO) can be increased [5].

The ANCA-associated vasculitis, in contrast, is pauci-immune with few or no immune deposits. Those antibodies may be specific for myeloperoxidase-anti-neutrophil cytoplasmic antibodies (MPO-ANCA) or proteinase 3-anti-neutrophil cytoplasmic antibodies (PR3-ANCA). The presentation of ANCA vasculitis is more systemic and virtually all organs can be affected. Renal involvement in ANCA-associated vasculitis is common and can range from asymptomatic relapsing hematuria with normal renal function to end-stage renal disease, presenting with nephritic syndrome features [6].

No optimal treatment strategy is established in this scenario but it seems reasonable to target the pathophysiology of both entities through precocious immunosuppression and plasmapheresis. A more attentive follow-up is required for patients with double-positive or single ANCA-positive disease because, contrarily to the anti-GBM disease, relapse is more frequent. Given so, even in anti-GBM patients that had negative ANCA titers at presentation, if a relapse becomes clinically apparent new serology must be obtained for confirmation. Concerning prognosis, interestingly, there appear to be no significant differences between mortality rates amongst these entities [1,2]. 

## Case presentation

A 61-year-old man was referred to the emergency department of our hospital by his assistant physician due to asthenia, nausea, and anorexia during the previous month coupled with rapidly deteriorating renal function. The past medical history included dyslipidemia, controlled arterial hypertension, partial thyroidectomy, and stable untreated psoriatic arthritis with no other known associated connective tissue or autoimmune conditions. The patient was also accompanied by a cardiologist due to a history of syncope but no chronic cardiomyopathy, arrhythmia, or coronary disease was found in the aetiologic investigation. The patient denied any knowledge of diabetes, stroke, or any infectious diseases. His family history was negative for thrombotic disorders as well as autoimmune conditions and otherwise unremarkable. He was on ivabradine 5 mg/day, telmisartan 80 mg + amlodipine 5 mg/day, and rosuvastatin 10 mg/day. No pharmacological allergies were known.

The patient did not present fever, weight loss, or arthralgia and no skin changes were apparent. Haematuria, dysuria, and foamy urine were not reported and the patient did not exhibit respiratory symptoms or hemoptysis. Eye redness/pain and headache were not mentioned and the patient denied mucous dryness or ulceration. No gastrointestinal symptoms were pointed out. No new drugs or toxic/herbs, namely herbal remedies or teas, were identified. The patient had no contact with domestic or farm/wild animals and had not traveled abroad recently.

On physical examination at admission, blood pressure was 174/86 mmHg, heart rate was 78 bpm with a rhythmic pulse, respiratory rate was 18/min, and the temperature was 36.5ºC. The air oxygen saturation was 94%. The pallor of the skin and mucous membranes was apparent. No anomalies were found in cardiopulmonary auscultation and the abdominal examination was unremarkable. No peripheral edema was found and capillary perfusion time was normal (<2 sec). The patient presented bilateral ankle swelling with no external inflammatory signs or pain, that he described as chronic and unaltered. Neurological examination at admission was normal.

Investigation

The most relevant performed laboratory studies and normal reference values are detailed in Table 1. A complete hemogram revealed mild anemia with normal white blood cells (WBC) and platelet counts; no schistocytes were found on peripheral blood smear ruling out, thrombotic microangiopathy coagulation tests were within normal range. Lactate dehydrogenase, total bilirubin, and haptoglobin were also normal, making hemolysis unlikely. C-reactive protein was only mildly elevated (23.1 mg/L). Creatine kinase and myoglobin levels were normal. Serum protein electrophoresis profile was typical and immunoglobin levels and light chain proteins in the serum were within normal range.

**Table 1:** Summary of laboratory findings ALP, alkaline phosphatase; ALT, alanine aminotransferase; aPTT: activated partial thromboplastin time; AST, aspartate aminotransferase; CK, creatine kinase; CRP, C-reactive protein; C3c, conjugated complement component 3; C4, complement component 4; FT4, free thyroxine; GGT, gamma-glutamyl transferase; LDH, lactate dehydrogenase; PTH, parathyroid hormone; TP, prothrombin time; TSH, thyroid-stimulating hormone

Parameter	Value	Ref. Value	Parameter	Value	Ref. Value
Hemoglobin	10.7 g/dL	12.0-16.0	TP	13 seg	10.1-13.6
Hematocrit	30.9%	37-49	aPTT	33.7 seg	24.2-36.4
Leucocytes	8.70 × 10^9 ^/L	4.0-11-0	AST	26 U/L	10-31
Platelets	260 ×10^9^/L	150-400	ALT	25 U/L	10-31
Haptoglobin	431 mg/dL	50 - 320	GGT	13 U/L	7-32
LDH	227 U/L	<225	ALP	73 U/L	30-120
CK	72 U/L	10-172	Total bilirubin	0.5 mg/dL	<1.2
Myoglobin	138 ng/mL	<146.9	TSH	3.21 μUI/mL	0.35-4.94
CRP	23.1 mg/L	<3.0	FT4	0.92 ng/dL	0.7-1.48
Creatinine	8.01 mg/dL	0.51-0.95	Serum iron	54 ug/dL	53 - 167
Urea	206 mg/dL	10-50	Ferritin	641 ng/mL	20-250
Uric Acid	8.7 mg/dL	3.6-8.2	Transferrin	159 mg/dL	200-360
Sodium	137 mEq/L	135-147	Vitamin B12	289 pg/mL	187-883
Potassium	5.6 mEq/L	3.5-5.1	Folic acid	4.3 ng/mL	2.2-17.5
Chlorum	106 mEq/L	101-109	25-OH-vitamin D	22 ng/mL	>30
Calcium (total)	2.5mmol/L	2.0-2.6	Proteins (urine)	0.7 g/L	<0.15
Magnesium	1.6 mEq/L	1.55-2.05	Erythrocytes (urine)	1016	<15
Phosphate	4.9 mg/dL	2.7-4.5	Leukocytes (urine)	20	<15
PTH	147 pg/mL	10-65	Pathological casts (urine)	Not detected	
25-OH-vitamin D	22 ng/mL	>30			
C3c	132 mg/dL	83-177			
C4	31 mg/dL	12 - 36			

Urea and serum creatinine were markedly elevated at admission (206 mg/dL and 8.01 mg/dL, respectively), but the electrolyte panel showed only slight hyperkalemia (5.6 mEq/L) and hyperphosphatemia (4.9 mg/dL) with no other deviations. Uric acid was also elevated. Mild elevation of parathyroid hormone (PTH) with low 25-OH-vitamin D and normal calcium suggested a subacute process. Complementary testing found no abnormalities in lipidic profile, cytocholestasis markers, thyroid function, serum iron, transferrin and ferritin, and vitamin B12 and folic acid levels.

The urinalysis showed marked erythrocyturia with slight proteinuria and leukocyturia that coupled with hypertension suggested a nephritic process but no pathological casts were found in urinalysis.

Regarding immunology markers, ANCA-MPO titer was 72 U/mL (normal value <20) and anti-GBM titer was 216.0 U/mL (normal value <7), both demonstrating a frank elevation. Serum tests for anti-streptolysin O, anti-nuclear antibodies, anti-cyclic citrullinated peptides, rheumatoid factor, anti-double-stranded DNA, anti-extractable nuclear antigens (ENA), and anti-cardiolipin antibody titers were negative and there was no complement consumption. Anti-treponemal tests, human immunodeficiency virus, hepatitis B virus, and hepatitis C virus serologies were also negative.

**Figure 1:**
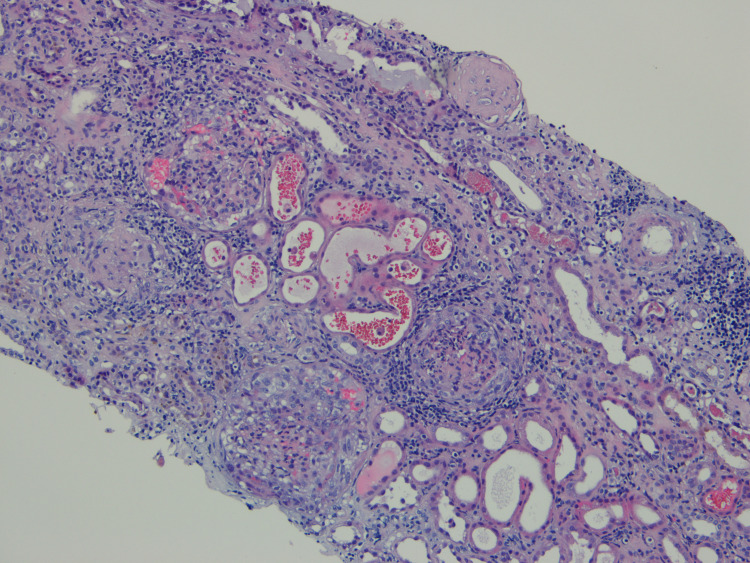
Kidney biopsy microscopy image Kidney biopsy microscopy photo (hematoxylin and eosin stain) showing glomeruli with crescents, mesangial proliferation, and sclerosis. Tubular atrophy and a hyaline cast are also apparent. Full biopsy comprised 21 glomeruli: five glomeruli were sclerosed, 10 glomeruli showed crescents (eight cellular and two fibrocellular crescents); the remaining glomeruli showed mesangial proliferation. There was a moderate degree of tubular atrophy associated with interstitial scarring and inflammation. Tubular necrosis and signs of hematuria were also present.

Given the positivity of ANCA-MPO and anti-GBM autoantibodies, renal biopsy was performed, confirming the diagnosis (Figure 1). The biopsy material allowed the observation of a total of 21 glomeruli with 47.6% of crescentic glomeruli (eight cellular crescents and two fibrocellular crescents). There were five sclerotic glomeruli and the remaining presented mesangial proliferation. There was also a moderate degree of tubular atrophy associated with interstitial scarring and inflammation and signs of haematuria as well. Immunofluorescence for immunoglobulin G (IgG) revealed linear deposits along the glomerular basement membrane and weaker staining in the tubular basement membranes, compatible with this entity. The lung CT showed no abnormalities.

Treatment

The patient was readily started on a high dose of methylprednisolone (1 g/day for three days) followed by a maintenance dose of 1 mg/kg of prednisolone. He was started on intravenous cyclophosphamide during the hospital stay and administered a cumulative dose of 5 g. Daily plasmapheresis with albumin reposition was initiated and continued for a total of 10 consecutive days and in alternate days for five more days. Renal function support with hemodialysis was needed shortly after diagnosis. *Pneumocystis jirovecii *prophylaxis with trimethoprim/sulfamethoxazole three times a week was also provided, as the patient was on high dose corticosteroids and immunosuppressant therapy.

Outcome and follow-up

The patient evolved with significant improvement of his systemic symptoms and despite maintaining adequate diuresis, renal function recovery was not obtained which motivated a regular hemodialysis program initiation. In what concerns antibody titers, steep descent in anti-GBM titers was noted at discharge (anti-GBM 7.9 U/mL) and ANCA-MPO titers normalized and both were persistently within normal levels during a follow-up of two years.

The patient was advised to seek medical attention immediately if abnormal symptoms presented to evaluate vasculitic relapse. Medical follow-up appointments were scheduled to assure an adequate multidisciplinary approach. Prednisolone was tapered and eventually stopped five months following hospital admission and there was no need for reinstitution.

Despite clinical and analytical stability, since the patient is hemodialysis-dependent (two sessions per week, three hours per session), a referral for a renal transplant was considered and is ongoing [7].

## Discussion

Acute kidney injury (AKI) includes an extensive differential diagnosis and an initial approach involves attempting to classify its etiology as prerenal (decreased renal perfusion pressure), intrinsic renal (involving the vessels, glomeruli, or tubulointerstitium), or postrenal (obstructive).

In the case of our patient, no renal or gastrointestinal losses were suspected and dehydration was not apparent at physical examination. There was no history of nonsteroidal anti-inflammatory drug usage and the chronically prescribed angiotensin II receptor blockers (ARB) had no chronological relation with the appearance of the AKI, making a prerenal etiology less likely.

Obstructive processes were excluded by the renal ultrasound and, therefore, an intrinsic renal etiology was suspected from an early stage. The patient did not present a urinary tract infection and no other infection site was suspected. A complementary broad investigation was requested and a double-positive anti-GBM and ANCA disease was ultimately confirmed.

Anti-GBM disease differential diagnosis involves alternative causes of acute glomerulonephritis potentially associated with pulmonary hemorrhage and other renal disorders in which linear IgG staining is seen. ANCA-positive vasculitides, immunoglobulin A (IgA) vasculitis/Henoch-Schönlein purpura, and mixed cryoglobulinemia syndrome, as well as systemic lupus erythematosus, are among the first group. These are potential causes of acute nephritides that can lead to pulmonary involvement either by pulmonary edema or direct vasculitic damage. The clinical distinction of such entities may be impossible, making serologic testing and kidney biopsy essential for a correct diagnosis.

Lung involvement was not apparent in the case of our patient. This can delay the diagnosis as the typical overt lung/kidney syndromic presentation is more clinically recognizable. Linear IgG staining on kidney biopsy can be seen in diabetic nephropathy and fibrillary glomerulonephritis but these can be easily distinguished from anti-GBM disease as serum anti-GBM antibodies are absent and other clinical and histological features are present.

According to one of the largest documented series, patients with double-positive anti-GBM and ANCA disease tend to present more evidence of chronic injury (sclerotic glomeruli/fibrosis) on renal biopsy compared to single anti-GBM disease. On the other hand, and quite surprisingly in the face of the previous information, double-positive patients tended to recover more often from dialysis-dependent renal injury after treatment but no overall survival differences were described. Disease relapse is far more common in ANCA-associated vasculitis and double-positive disease than in single anti-GBM disease. This entity appears to express a hybrid phenotype, justifying a more aggressive approach in precocious settings for anti-GBM disease and careful long-term follow-up/immunosuppression for the ANCA positive features [1].

This case comes across as a learning opportunity as the patient expressed subacute disease with systemic symptoms, frequently associated with systemic ANCA-associated vasculitis, and an AKI requiring hemodialysis, more characteristic of the anti-GBM disease. Chronic kidney injury features were apparent in histological examination and the instituted treatment was multimodal to ensure the approach of both pathophysiological entities.

## Conclusions

Anti-GBM disease and MPO-ANCA-associated vasculitis are small vessel vasculitides that can prompt rapidly progressive glomerulonephritis and alveolar hemorrhage with a risk of organ failure and death. Despite the rareness of each of these entities, the overlap ANCA and anti-GBM displays a higher frequency than determined by chance and it might influence disease onset and course. No optimal treatment strategy is defined but targeting the pathophysiology of both entities through precocious immunosuppression and plasmapheresis seems a reasonable and effective approach. There appear to be no significant differences between mortality rates among these entities.
